# A Quantitative Comparison of Slackline Balancing Capabilities of Experts and Beginners

**DOI:** 10.3389/fspor.2022.831362

**Published:** 2022-03-10

**Authors:** Kevin Stein, Katja Mombaur

**Affiliations:** ^1^Optimization, Robotics and Biomechanics, Institute of Computer Engineering at Heidelberg University (ZITI), Heidelberg University, Heidelberg, Germany; ^2^Canada Excellence Research Chair in Human-Centred Robotics and Machine Intelligence, Departments of Systems Design Engineering & Mechanical and Mechatronics Engineering, University of Waterloo, Waterloo, ON, Canada

**Keywords:** slackline, balance, performance indicators, kinematic, analysis

## Abstract

Mechanical stability criteria are able to explain balance and robustness during simple motions, however, humans have learned many complex balancing tasks for which science lacks a thorough understanding. In this work, we analyzed slackline balancing to define general balance performance indicators. The goal is to not only measure slackline expertise, but to be able to quantify stability during any balance task. For this, we compared beginners that had never balanced on a slackline before to professional slackline athletes. Further, all participants performed a static balance test, based on which we divided beginners into a balance-experienced and a balance-inexperienced group. On average, the balance experienced group was able to balance twice as long on the slackline and therefore, we showed that this static balance experience is a predictor of slackline balance performance. Based on over 300 balancing trials on the slackline of 20 participants, we then defined and evaluated over 30 balance metrics. The parameters can be grouped into quantification of stability and recovery movements, balance specific skills and balance strategies. We found that normalized angular momentum and center of mass acceleration are measures for overall stability, with lower values representing better stability and fewer recovery movements. We showed that improved hand coordination and adjusted stance leg compliance are valuable skills for balance tasks. especially when controlling external forces. Looking at posture and movement strategies, we found that professional slackliners have adapted a different mean pose with larger inertia and an upright head position, when compared to beginners.

## 1. Introduction

**Figure 1:**
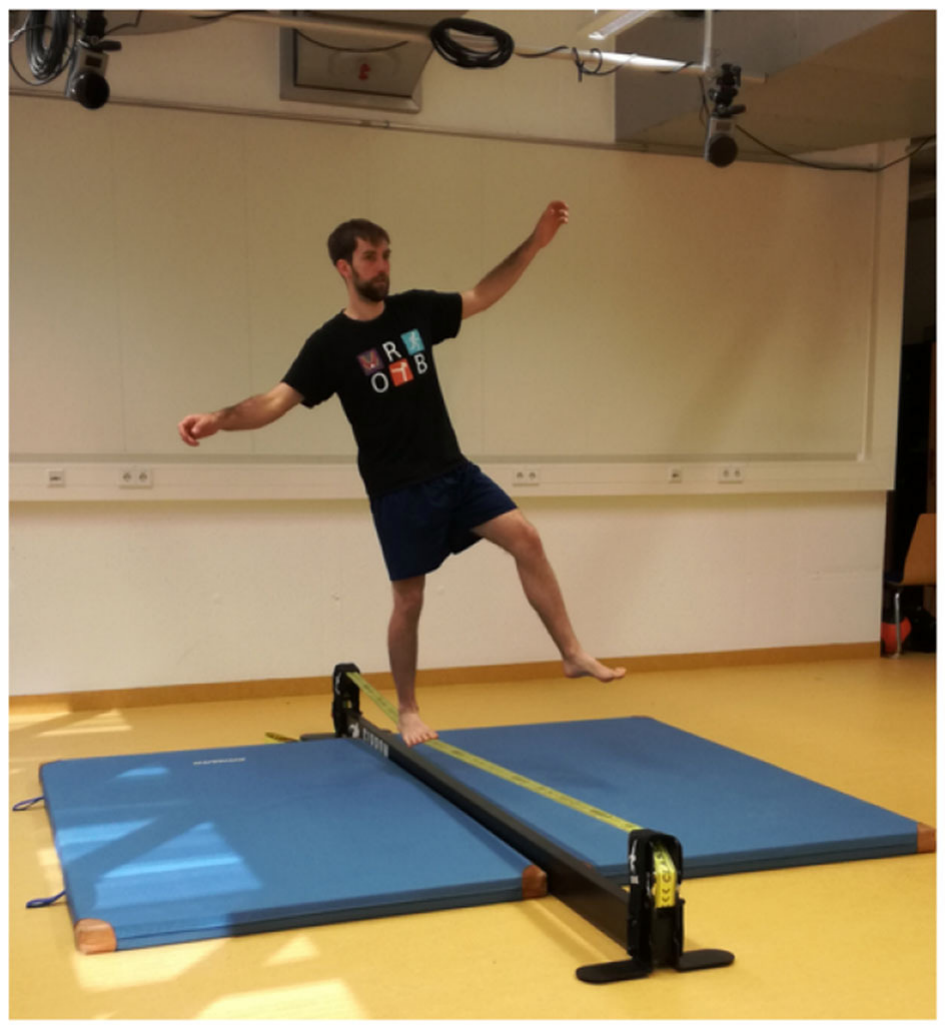
Slackline balancing.

In challenging balance exercises signs of stable or impaired balance are much more obvious than in every day situations. Therefore, a lot can be learned for balance training, quantification of stability and even humanoid robotics by analyzing those tasks. Slackline balancing is a sport where the athlete tries to maintain balance on an elastic ribbon band that is mounted between two anchor points as shown in Figure 1. Unlike balancing on flat surface, the contact point with the slackline can swing both sideways and vertically, which increases the difficulty of maintaining an upright position. The restoring forces always point toward the straight line defined by the two anchor points (Paoletti and Mahadevan, 2012; Athanasiadis, 2017). The subject's Center of Mass (CoM) is well above the anchor points, thus the Virtual Pivot Point (Maus et al., 2010), which is the point where all forces acting on the subject coincide, is below the CoM and does not provide stabilization, but makes the system intrinsically unstable. So far only a few studies are available on slackline balancing: Training effects of slackline balancing on posture, neuromuscular performance and other balance tasks have been well-studied (Donath et al., 2013, 2015). They found task specific improvements but limited transfer to other balance tasks. Keller et al. found improved postural control and reduced h-reflexes (Keller et al., 2011). These reflexes are responsible for the shaking knee movement a beginner experiences when trying to stand on a long slackline for the first time. Little research can be found on the question how to evaluate slackline expertise and compare balance performance. Kodama et al. (2017) analyzed one beginner and one expert and found differences in hand coordination and less knee and CoM variability. Serrien et al. (2016) and Serrien et al. (2017) employed self-organizing maps to analyze kinematic motion capture before and after a 6 weeks training intervention. They found that the balance coordination pattern changed significantly by means of increased range of motion and decreased velocity in joints.

Our research aims at defining quantitative and standardized stability metrics that allow us to quantify stability in a general way and analyze the effect of balance training. We define and demonstrate them in context of slackline balancing, however they can be applied to any kind of balance task. In previous work we proposed normalized angular momentum, CoM acceleration, kinetic energy and stance foot acceleration as performance indicators for slackline balancing (Stein and Mombaur, 2019). We analyzed single leg slackline balancing and slackline walking from a total of 11 participants divided into three groups. Subjects that had never done slackline balancing before were grouped into a beginner and a sportive, balance-experienced, beginner group depending on their sports experience. The professional group consisted of slackline athletes that practiced the sport regularly. We published overviews of the analyzed performance indicators and formulated hypothesis on crucial group differences, however, no statistical analysis was performed since there were too few participants per group. In this article, we publish the final results of the study and present the following new contributions:

- A total of 20 participants and 300 trials of slackline balancing are analyzed.
- All participants performed a static balance pre-test that allows us to objectively group beginner subjects into a balance-experienced (sportive) and balance-inexperienced group.
- We present a clear definition and evaluation of new performance indicators.
- We perform a statistical evaluation of all slackline balance performance indicators and group comparison.

## 2. Methodology

### 2.1. Balance Assessment

We recorded marker based motion capture data of 6 professional slackliners (2 female, 4 male) and 14 beginners, that had never balanced on a slackline before (4 female, 10 male). Age was between 18 and 32 years. Weight was 72(11) kg, height 1.76(10) m, and BMI 23(2) kg m-2.

#### 2.1.1. Lab Protocol and Motion Recording

Participants were invited to the motion capture lab of the Heidelberg Center for Motion Research (HCMR). Written informed consent was obtained from all subjects before the measurements. The protocol consisted of subject preparation, a static balance test and two rounds of slackline balancing with a 5 min break in between, all of which will be described in the following paragraphs.

#### 2.1.2. Subject Preparation

We measured the height and weight of the subject and placed 51 retro-reflective motion capture markers. Forty-nine markers are from the Gait-IOR marker set guidelines (Leardini et al., 2011) and two additional markers are placed at the medial epicondyle of the humerus for better upper arm and shoulder angle reconstruction. The marker set consists of 45 tracking markers and 6 static markers. Static markers were only recorded once, during the static trial to define the subject-specific rigid body model and were removed afterwards.

#### 2.1.3. Static Balance Pre-test

All subjects performed a static balance test. It consists of the following five tasks that were done in sequence:

(1) Close Parallel Stance: Standing on both feet, with the feet close together.
(2,3) Single Leg Stance: Standing on one foot, once for each foot.
(4,5) Tandem Stance: Standing with the feet aligned and the heel of one foot in touch with the toes of the other, once for each foot in front.

The total time for each task was 1 min. We placed tape marks on the floor to ensure correct foot placement. After 30 s in each task participants were instructed to close their eyes and balance for another 30 s. They were instructed to maintain their hands on the pelvis and remain in the pose for as long as possible. A step or arm movement should only occur to prevent falling. In case of a fall during the eyes open situation, subjects were asked to re-assume the pose and continue balancing. If they fell after closing their eyes we continued with the next task. A 30 s break was given between each static balancing task. Subjects started outside the marked area, walked into the position at their own speed and placed the hands on the pelvis when they were stable. The motion capture recording was then started. After 60 s participants were instructed to step out of the marked area and the recording was stopped. For single leg balancing and the tandem stance, the (leading) foot for the first trial was chosen at random and switched for the second trial.

#### 2.1.4. Slackline Balancing

The slackline was installed using the Gibbon Slackrack 300 (ID Sports GmbH, Gibbon Slacklines, Stuttgart, Germany). It measured 3 m in length, 5 cm in width and was mounted 31 cm above the ground. The motions were recorded using the marker-based motion capture system Qualisys consisting of 8 Oqus 500 cameras at a frame rate of 150 Hz. We recorded:

- **Single Leg Slackline Balancing**: Balancing on one foot in the middle of the slackline, left and right leg interchanging.
- **Tandem Stance Slackline Balancing**: Balancing on both feet in the middle of the slackline, leading foot interchanging.
- **Walking on the Slackline**: Beginning from one end of the slackline and walking to the other end. Then walking backwards to the start, going back and forth.

Subjects were asked to maintain balance for as long as possible. After falling, the same kind of slackline balancing was repeated after a short break. Up to 10 trials were recorded and then continued with the next balance configuration. A 5 min break was given after each kind of balancing was performed once and then the whole slackline balance protocol was repeated a second time. The slackline balance experiments were approved by the ethics committee of the Faculty of Behavioral and Cultural Studies of Heidelberg University according to the Helsinki Declaration (AZ Mom 2016 1/2-A1, 2016 with amendment 2019).

### 2.2. Evaluation

#### 2.2.1. Static Balance Test Evaluation

We first evaluate the static balance test to group beginner participants into a balance-experienced and balance-inexperienced group. Motion capture recordings were cut from the beginning of each task till the point where the subject closed the eyes and again after 1 min, resulting in a total of 10 static balance recordings per participant. For each part we assessed the time in balance defined as the time until either a step occurred, the hands were taken of the pelvis, or the stance foot was shifted. From this we compute the total time in balance and summed the times of corresponding foot conditions. We get the time in balance for the following situations: parallel stance with eyes open, parallel stance with eyes closed, single leg stance with eyes open, single leg stance with eyes closed, tandem stance with eyes open and tandem stance with eyes closed. We will use Spearman's rank correlation (Kendall, 1948) to see how subjects time in balance on the slackline correlates to the times in balance in the static balance test.

#### 2.2.2. Biomechanical Evaluation of Motions on the Slackline

**Figure 2:**
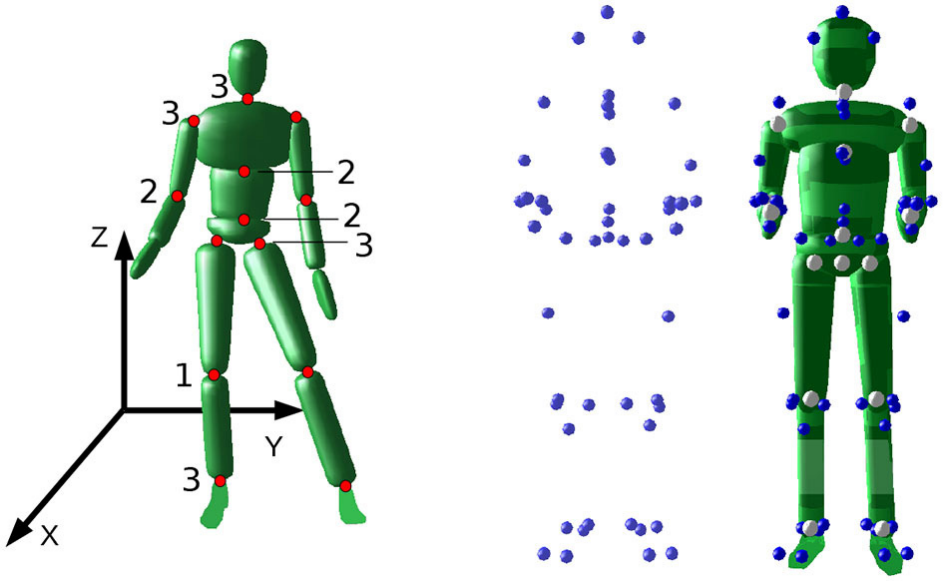
Left: The model with the location of the joints, the number DoF, and the coordinate system. Middle: The marker recording of the static pose of the subject. Right: The rigid body model derived from the static trial.

We analyze the marker motion capture data based on subject-specific rigid-body models. The model consists of 16 bodies, 15 joints and is based on the anthropomorphic data by De Leva (1996). The arms, the legs, and the trunk consist of three rigid-bodies each. Head and Neck are modeled as a single body. The length of each segment and the joint center locations are estimated from the static trial recording and the measured subject height based on the work by Leardini et al. (2011), Cappozzo et al. (1995), and Rab et al. (2002). The hip joints are estimated following the pelvis model by Bell et al. (1989). Neck, shoulder, hip and ankle joints are modeled as spherical joints with 3 Degrees of Freedom (DoF). Lumbar, thorax and elbow joints are modeled with 2 DoF each and the knee joints with one DoF. A 6 DoF floating base with three translational and three rotational DoF is attached to the Pelvis segment. The dynamic properties of each segment are taken from De Leva (1996). Figure 2 shows the model and number of DoF at the left, the static trial in the middle and the model fitted to the static trial at the right. We use Puppeteer (Felis, 2015) to place virtual markers on the rigid body model according to the static trial recording.

Joint angles of all marker recordings were computed using also Puppeteer which employs an iterative Levenberg-Marquardt algorithm (Levenberg, 1944). Joint angle trajectories are filtered using a 5^*th*^-order Butterworth filter with a cutoff frequency of 9 Hz. Joint velocities and accelerations were computed using numerical differentiation. All slackline balance recordings were cut from the time the subject left contact with the floor and until the time the foot left contact with the slackline.

### 2.3. Slackline Balance Performance Indicators

In the following, we propose performance indicators for slackline balancing and discuss how they relate to balance control. Most performance indicators can be computed for standing and walking. Additional analysis that requires a stance and a swing leg is only well-defined for single leg balancing when there is no double support phase. Parameters are grouped into *Quantification of Stability and Recovery Movements, Balance Specific Skills*, and *Balance Strategies*.

#### 2.3.1. Time in Balance

For slackline balancing, the time a person is able to maintain balance is arguably the most evident performance indicator. Experts are able to balance for more than 2 min. We found that talented beginners are able to balance up to 1 min by the end of their first ever slackline session in our lab.

#### 2.3.2. Quantification of Stability and Recovery Movements

##### 2.3.2.1. Normalized Angular Momentum / Average Angular Velocity

**Figure 3:**
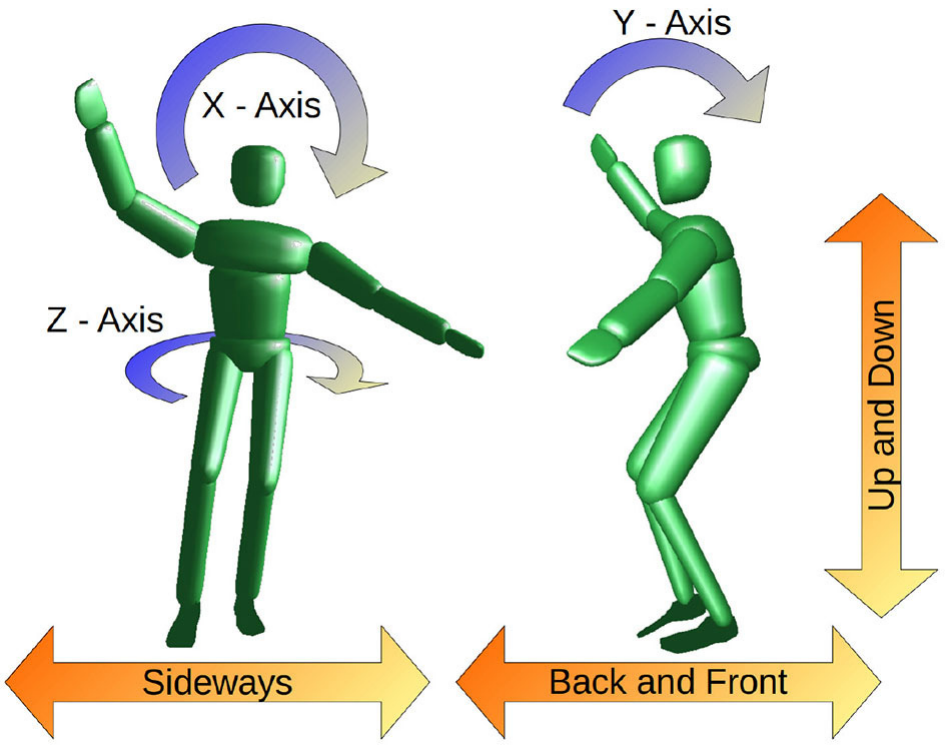
Illustration of CoM movement (orange) and average angular velocity (blue) in the different directions.

We normalize the angular momentum around the subjects CoM to find the average angular velocity as suggested by Essén (1993).


(1) $$\begin{array}{r} \omega \left(t\right)=I{\left(q\left(t\right)\right)}^{-1}L\left(q\left(t\right),\overset{∙}{q}\left(t\right)\right) \end{array}$$

where ***I***(***q***) is the total inertia of the rigid body system for a given joint configuration at time *t*. This prevents bias from differences in subject weights and heights. In terms of balance performance indicators, the average angular velocity tells us how fast the subject rotates around the specific axis. For stable balancing, the mean angular velocity is zero, and therefore we can use the RMS to quantify how much rotational movement is present during a given motion. We can analyze rotation around the three coordinate axes separately, as it is visualized in Figure 3:

- Around the axis of the slackline (X-Axis):We expect the main balancing movement around this axis to counteract the instability introduced by the slackline. Larger values represent more and greater recovery movements.
- Around the axis perpendicular to the Slackline (Y-Axis):We assume that experienced subjects are able to maintain an upright position and do not tilt back and forth. This should be the case for single leg balancing and walking.
- Around the vertical axis (Z-Axis):We expect experts to maintain an upper body orientation perpendicular to the slackline. Turning parallel to the slackline increases the difficulty dramatically and is therefore not desired.

##### 2.3.2.2. Center of Mass Dynamics and Slackline Interaction

The foot contact with the slackline is able to swing sideways and in up and down direction. The contact forces are constantly changing and the stance foot of the subject is being accelerated by the spring-like slackline (Paoletti and Mahadevan, 2012; Athanasiadis, 2017). We suggest the following performance indicators related to the CoM dynamics:

- Root mean squared (RMS) of CoM accelerationThe mean CoM acceleration is zero for stable balancing, hence, a lower RMS value represents less overall acceleration. We analyze movement perpendicular to the slackline, along the slackline and in vertical direction separately. During the walking tasks, less CoM acceleration, results in a smoother walking motion. Overall we claim that this resembles better balance control.
- RMS of CoM support polygon projectionStatic balance requires the CoM ground projection to be within the support polygon. A larger distance to the edges of the support polygon throughout a movement is associated with better stability. We can compute the variation of the CoM position in the support polygon as performance indicator.
- Mean CoM velocity during walkingExperts should be able to walk at a higher velocity when compared to beginners.

##### 2.3.2.3. Normalized Kinetic Energy

Kinetic energy quantifies the overall movement and combines CoM velocity and angular velocity. However, this quantity depends on the total subject mass. To allow for an approximate comparison between persons of different mass we propose to normalize the kinematic energy by dividing through the subject mass. For single leg balancing, where the intended pose requires no kinetic energy at all, we expect higher kinetic energy for beginner subjects as they are expected to perform more movement to maintain balance. We compute the mean of normalized kinetic energy *E*_*kin*_ over one trial as performance indicator.

##### 2.3.2.4. Balance Energy Ratio

For slackline walking, we define the balance energy ratio *R*_*balance*_ that measures how much of the kinetic energy is due to translational movement and how much is used to maintain balance. We define the normalized translational kinetic energy as the CoM velocity ċ squared and the balance energy ratio as the fraction of the kinetic energy that is not in translational movement:


(2) $$\begin{array}{r} R_{balance}\equiv \frac{E_{kin}-\frac{1}{2}{\overset{∙}{\text{c}}}^{2}}{E_{kin}} \end{array}$$

For walking tasks we expect professional slackline athletes to walk more efficiently by means of a lower balance energy ratio. We compute the ratio for every instance of the trial and take the mean as performance measure.

#### 2.3.3. Balance Specific Skills

##### 2.3.3.1. Movement Coordination

**Figure 4:**
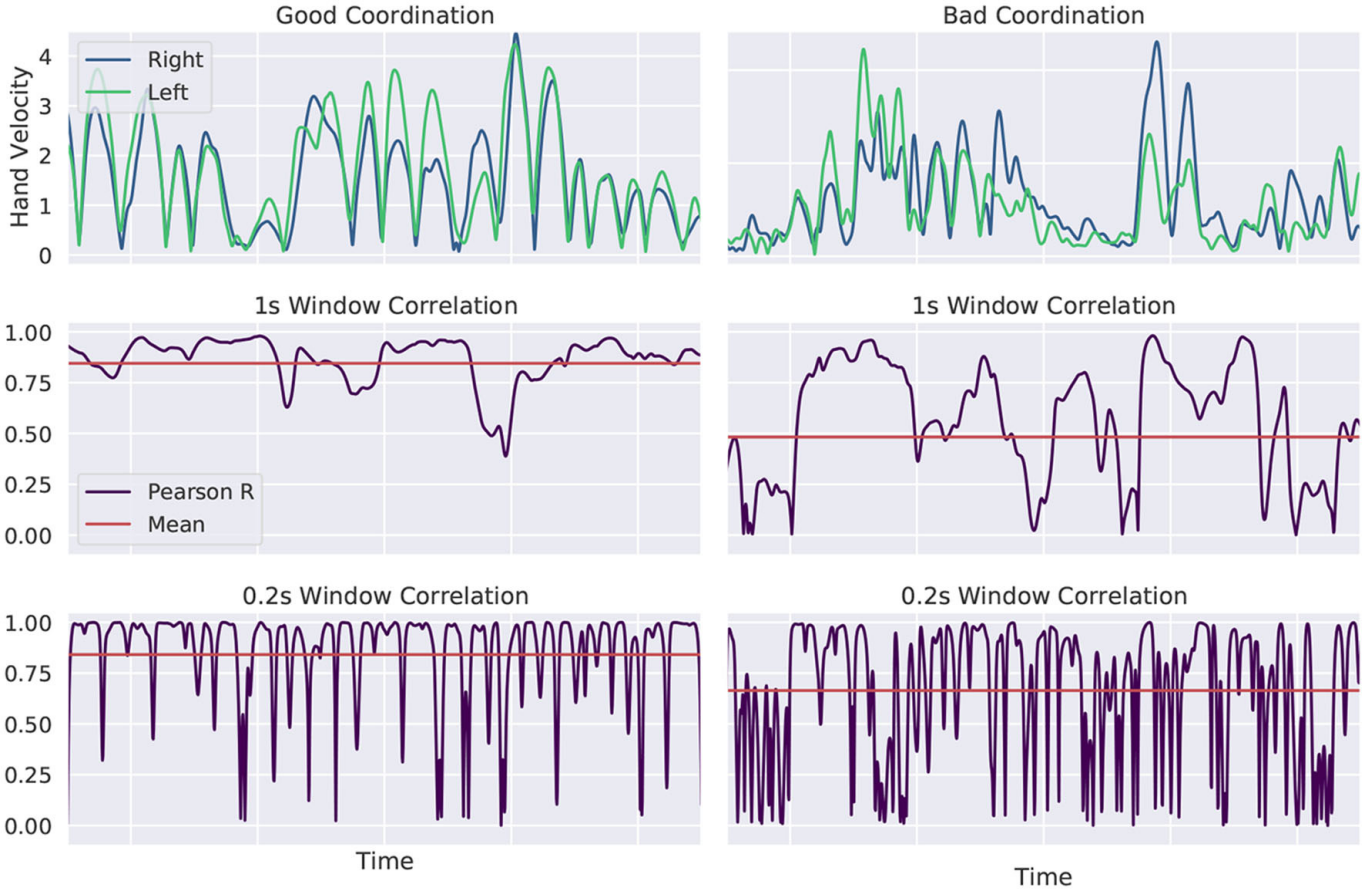
Evaluation of hand movement coordination. Hand velocities are shown at the top for well and badly coordinated movement. The rolling window correlation is plotted for a 1 s window in the middle row and for a 0.2 s window in the bottom row. The intended relationship between the mean value and the similarity of hand velocity trajectories can be observed. More similar hand velocities result in a larger value for the proposed performance indicator.

We expect controlled and well-coordinated hand movement as a sign of good balance control. This was already found by looking at the hand position of two individuals of different skill levels in Stein and Mombaur (2019) and Kodama et al. (2017). In this work, we propose a single quantifiable measure for hand movement coordination. We compute the rolling window Pearson correlation between the absolute hand velocity values of the left and right hand, similar to algorithms developed by Tschacher and Meier (2020) or Cheong et al. (2020). We take a small subset of the whole trajectory of a given window length and compute the “local” Pearson correlation (Kirch, 2008). The window is then shifted by one data point and the correlation computed for the new subset. This is done for the entire trajectory. The performance indicator for movement coordination is defined as the mean of all correlation values. We evaluate short term movements of 0.2 s and longer periods of 1 s. A sample evaluation is shown in Figure 4. Well-coordinated hand movement is shown in the left column, arbitrary movement in the right column. Hand velocities are plotted in the top row. We see overlapping trajectories at the left, as it is expected for good coordination and independent hand movement at the right. The rolling window correlation for a 1 s window is plotted in the middle row and for a 0.2 s window in the bottom row. The mean values, that we intend to use as performance indicator, are plotted for both measurements in red. The intended relationship between the mean value and the similarity of hand velocity trajectories can be observed.

##### 2.3.3.2. Stance Leg Compliance

For this performance indicator we evaluate the acceleration of the stance foot and compare it to the acceleration at the subjects CoM. We compute the correlation between the stance foot acceleration and the CoM acceleration over the whole trajectory. Adjusted compliance in the stance leg would allow the athlete to absorb the changing interaction forces with the slackline.

#### 2.3.4. Balance Strategies

We analyze posture and joint movement. Consistent behavior of the professional group could reveal strategies that then can be communicated to beginners. We define the utilized range of motion as the standard deviation of the joint angle over the whole joint angle trajectory. This can give us insights in which joints are mainly used for the balance task and which joints do not contribute. We expect to find a larger value for the utilized range of motion in professional slackliners. Beginners might only be stable in some poses and fall when it comes to more difficult situations, therefore showing a lower value.

#### 2.3.5. Evaluation Pipeline

- **Recording**: Kinematic data and the static trial are recorded following the protocol of the study.
- **Modeling and Kinematic Fitting**: The subject-specific model is created from the static trial. Joint angle trajectories are computed based on the inverse kinematics using Puppeteer (Felis, 2015).
- **Computation of raw Performance Indicator Trajectory**: All performance indicators were computed using the RBDL (Felis, 2016). The library already supports the computation of CoM dynamics, Angular Momentum and the ZMP position. We implemented functions that compute the inertia, the normalized angular momentum and hand coordination.
- **Summary and Statistics**: We summarize the performance indicator into one single value per trial. This can be the mean or standard deviation. Groups are compared using a Wilcoxon Rank-Sum Test (Mann and Whitney, 1947) and Cohen's-d (Cohen, 2013).

## 3. Results and Discussion

### 3.1. Static Balance Pre-test and Subject Grouping

**Figure 5:**
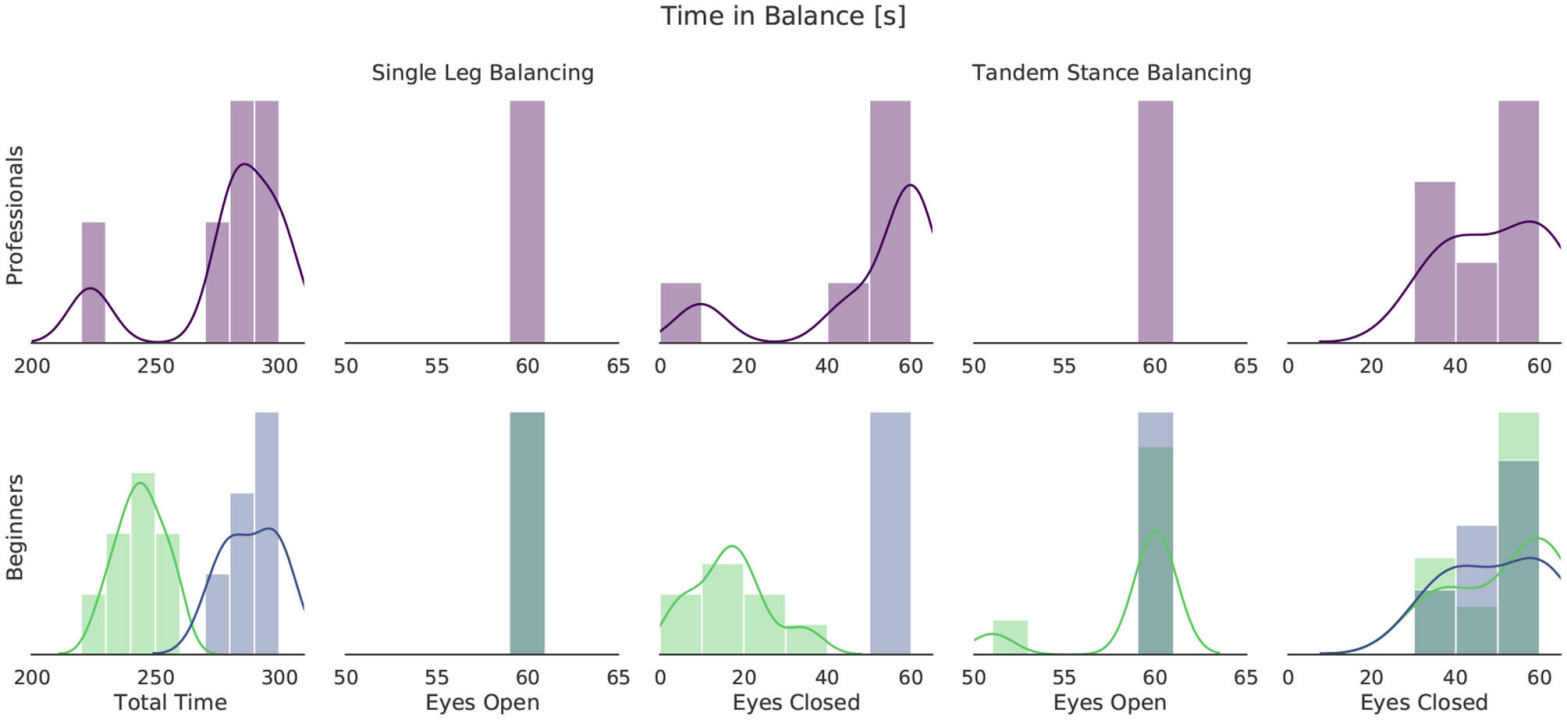
Histogram of time in balance during the static balance test for all participants of the slackline study. Professional slackline athletes are shown at the top, beginners at the bottom. A threshold of 260 s of total balance time was chosen to divide the beginner group at the bottom. Balance-inexperienced beginners are shown in light green, balance-experienced (sportive) beginners in blue.

Figure 5 shows histograms of the time in balance for professional slackliners at the top and for beginners at the bottom. The summed time of all 10 tasks of the test is shown at the left. A maximum of 300 s could be achieved. The right four plots show the combined time in balance for single leg balancing and tandem stance with eyes open and eyes closed. Since every participant managed the full 60 s standing on both legs, this plot is not shown. Beginners and professional slackliners show similar distributions for tandem stance and single leg stance with eyes open. Differences between the groups are found for single leg stance with eyes closed. Almost 50 % of the beginners failed to maintain balance throughout the entire task, whereas most of the professional slackliners managed. This difference is also visible in the total time. There are two clusters for the beginner group, depending on whether they managed the single leg balancing with eyes closed or not. Following the data, we define a threshold of 260 s of total balance time to divide the beginners into six balance-experienced, sportive, beginners and eight balance-inexperienced beginners. In the following, and in upcoming figures, we refer to the balance-experienced group as sportive group and to the balance-inexperienced group as beginner group for better readability.

#### 3.1.1. Connection Between Static Balance and Balancing on the Slackline

**Figure 6:**
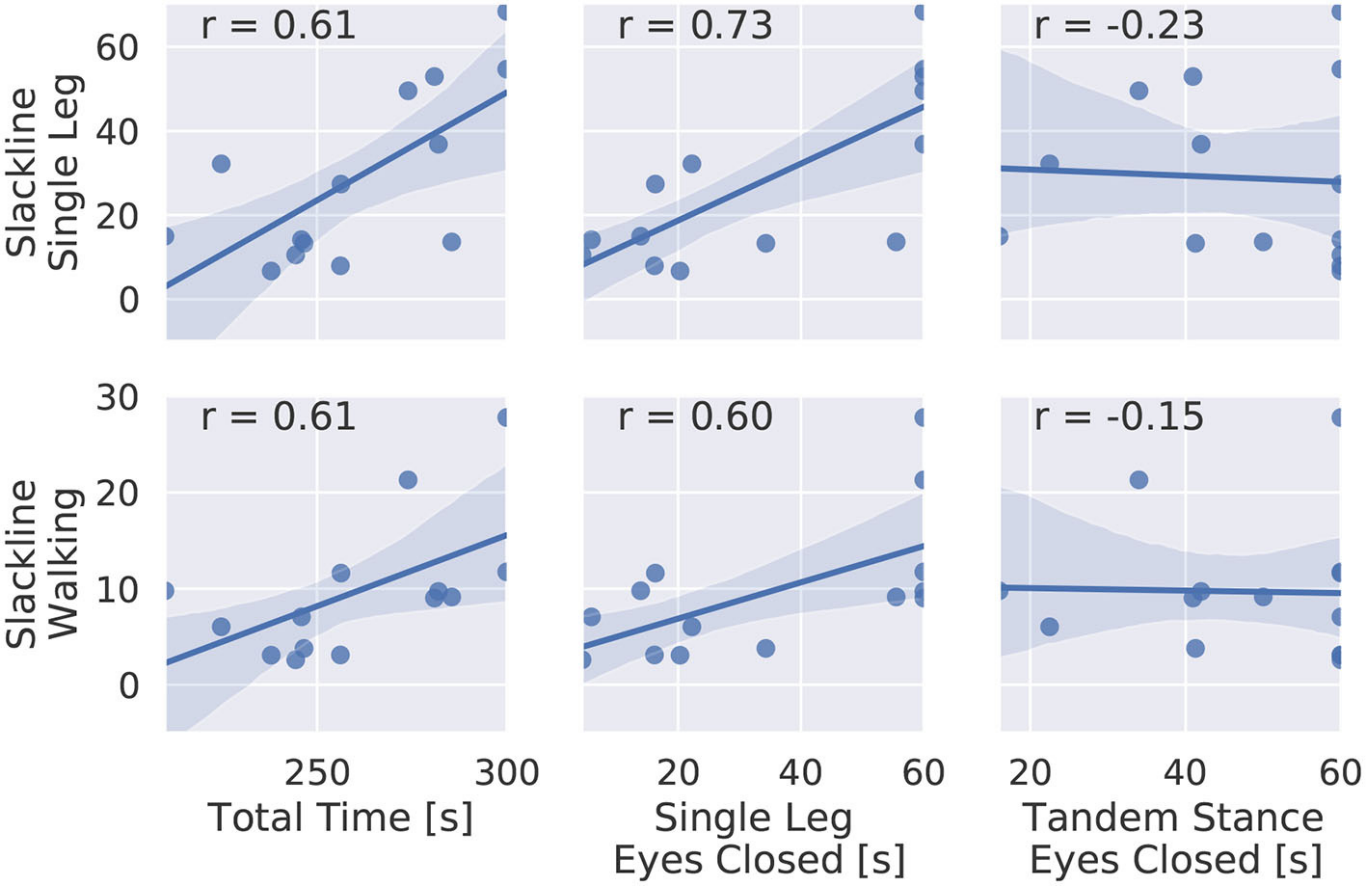
Scatter plot of balance time on the slackline and time in balance during the static balance test for all beginner subjects. Spearman's rank correlation is given.

Figure 6 shows the average time in balance on the slackline per beginner subject plotted against the total time in balance of the balance test. We see large correlation (*r*>0.6) for the total time and the single leg case with eyes closed for both slackline tasks. There is no correlation to tandem stance balancing. We propose that static balance capabilities, by means of balancing on one leg with eyes closed, can be used as a predictor for slackline balance performance.

In Table 1, we summarize all slackline recordings of the study per group. We consider trials with less than 8 s as unstable and exclude them from further analysis, since we would mainly evaluate random and uncontrolled behavior. Counts vary due to different number of participants per group and skill level. For all groups, we find that single leg balancing shows the highest success rate and tandem stance is the most difficult task. Beginners struggle to walk and to perform the tandem stance leading to a low percentage of trials that we can evaluate (23 % for walking and 13 % for the tandem stance). Professionals balanced for longer time and therefore showed fatigue, leading to fewer trials that were recorded per participant. Due to the few valid beginner trials for tandem stance, we only evaluate the performance indicators for single leg balancing and walking.

**Table 1:** Overview of study participants.

**Group**	***N***	**Single leg**	**%**	**Walking**	**%**	**Tandem stance**	**%**
Beginners	8	96 / 160	60	33 / 148	23	17 / 132	13
Sportive	6	76 / 85	90	56 / 83	67	35 / 70	50
Professional	6	43 / 43	100	34 / 36	94	24/ 30	80

*The number of recorded and evaluated trials is shown for single leg balancing, walking, and tandem stance*.

### 3.2. Performance Indicators for Slackline Balancing

Tables 2, 3 summarize all performance indicators. They show the mean value, standard deviation and ranges, for each group. In Tables 4, 5, we compare the groups using Cohen's-d (Cohen, 2013) and the Wilcoxon Rank-Sum Test (Mann and Whitney, 1947). In this section we discuss the main findings in more detail.

**Table 2:** Summary of all performance indicators for single leg balancing.

	**Beginners**				**Sportive**				**Professional**			
**Variable**	**Mean** **±SD**		**Range**		**Mean** **±SD**		**Range**		**Mean** **±SD**		**Range**	
Time [s]	14.27	14.15	0.67	73.33	42.32	35.44	3.25	133.62	72.31	42.51	14.53	190.23
Head orientation [°]	-18.00	11.80	-45.78	-0.96	-19.74	14.99	-50.60	0.24	-6.78	3.46	-13.63	2.84
Mean frontal shoulder angle [°]	66.56	22.57	25.54	122.76	56.67	20.27	21.32	114.57	87.96	12.94	51.19	109.64
Mean elbow angle [°]	52.54	16.07	19.79	86.21	64.18	13.25	21.73	97.45	35.85	8.12	17.86	52.75
Utilized elbow angle [°]	11.84	4.66	0.92	26.06	12.21	6.36	2.43	31.21	16.25	4.57	8.66	25.36
Utilized frontal shoulder angle [°]	19.11	9.79	1.99	62.54	18.92	10.57	2.67	65.67	29.48	8.75	14.08	46.24
Hand coordination (1.0s)	0.62	0.08	0.42	0.82	0.71	0.08	0.52	0.85	0.70	0.06	0.58	0.82
Hand coordination (0.2s)	0.73	0.03	0.64	0.79	0.76	0.03	0.69	0.83	0.77	0.02	0.73	0.81
Normalized angular momentum X [° s-1]	23.26	7.12	6.24	61.06	20.31	4.34	10.23	31.01	19.38	4.85	12.09	37.35
Normalized angular momentum Y [° s-1]	5.54	3.22	1.22	20.31	4.74	2.46	1.31	14.66	4.57	1.57	1.47	7.96
Normalized angular momentum Z [° s-1]	12.30	7.72	1.93	49.34	11.16	6.73	2.47	39.62	8.70	2.70	4.61	16.39
Stance foot acceleration Y [m s-2]	1.70	0.88	0.46	4.36	1.44	0.57	0.52	3.02	1.26	0.42	0.67	2.63
Stance foot acceleration Z [m s-2]	0.62	0.25	0.34	1.90	0.49	0.18	0.21	0.96	0.50	0.17	0.18	1.02
CoM acceleration X [m s-2]	0.16	0.06	0.06	0.41	0.12	0.04	0.06	0.23	0.13	0.05	0.08	0.34
CoM acceleration Y [m s-2]	0.34	0.11	0.10	0.73	0.29	0.08	0.14	0.51	0.28	0.08	0.16	0.56
CoM acceleration Z [m s-2]	0.43	0.16	0.10	0.93	0.28	0.10	0.13	0.56	0.26	0.07	0.13	0.49
Utilized knee angle [°]	3.60	1.76	0.69	10.98	4.02	1.76	1.12	8.56	4.72	2.41	1.33	11.58

*For each group the mean value, standard deviation, and ranges are given*.

**Table 3:** Summary of all performance indicators for walking on the slackline.

	**Beginners**				**Sportive**				**Professional**			
**Variable**	**Mean** **±SD**		**Range**		**Mean** **±SD**		**Range**		**Mean** **±SD**		**Range**	
Time	5.41	4.23	0.67	27.19	14.02	11.42	1.69	67.69	67.21	42.99	7.73	188.27
Head orientation [°]	-35.36	7.60	-47.73	-5.31	-27.13	17.05	-54.66	-0.56	-9.46	6.65	-26.14	-3.12
Mean frontal shoulder angle [°]	74.11	15.47	50.75	124.16	65.59	15.97	31.09	108.72	93.50	16.50	48.21	125.59
Mean elbow angle [°]	54.48	14.81	26.45	86.27	60.77	12.77	42.36	86.50	30.34	13.34	8.58	49.04
Utilized elbow angle [°]	13.63	3.94	7.86	23.15	15.29	6.35	6.94	30.78	17.21	4.40	8.60	25.95
Utilized frontal shoulder angle [°]	28.14	13.73	11.76	70.26	28.09	15.62	5.70	70.02	28.19	11.10	13.79	57.37
Hand coordination (1.0s)	0.59	0.10	0.38	0.78	0.70	0.07	0.54	0.90	0.75	0.07	0.64	0.88
Hand coordination (0.2s)	0.73	0.05	0.59	0.80	0.76	0.03	0.68	0.85	0.78	0.02	0.75	0.84
Normalized kinetic energy [J kg-1]	0.12	0.05	0.04	0.21	0.08	0.04	0.03	0.18	0.06	0.01	0.04	0.10
Energy ratio	0.59	0.09	0.39	0.73	0.68	0.14	0.41	0.93	0.65	0.08	0.49	0.78
Normalized angular momentum X [° s-1]	24.92	5.89	13.11	38.91	24.19	6.21	12.96	44.16	21.57	3.05	16.58	28.02
Normalized angular momentum Y [° s-1]	14.55	3.10	8.58	21.82	10.26	3.44	4.49	17.26	8.50	1.42	6.04	11.18
Normalized angular momentum Z [° s-1]	26.30	9.25	8.03	44.98	21.89	9.10	7.71	43.48	16.51	4.25	10.17	28.35
CoM acceleration X [m s-2]	0.41	0.13	0.18	0.66	0.33	0.16	0.16	0.96	0.30	0.08	0.18	0.45
CoM acceleration Y [m s-2]	0.44	0.10	0.24	0.71	0.39	0.09	0.23	0.63	0.31	0.05	0.21	0.39
CoM acceleration Z [m s-2]	0.66	0.23	0.30	1.23	0.49	0.16	0.24	0.91	0.41	0.08	0.25	0.58
Walking speed [m s-1]	0.26	0.08	0.12	0.46	0.16	0.10	0.02	0.40	0.15	0.04	0.09	0.25

*For each group the mean value, standard deviation, and ranges are given*.

**Table 4:** Group comparison of all performance indicators for single leg balancing.

	**Beginner/Sportive**		**Beginner/Professional**		**Sportive/Professional**	
**Variable**	***p*-val**	**d**	***p*-val**	**d**	***p*-val**	**d**
Time [s]	**<0.0001**	−1.17	**<0.0001**	−2.47	**<0.0001**	−0.78
Head orientation [°]	0.80	0.13	**<0.0001**	−1.12	**<0.001**	−1.06
Mean frontal shoulder angle [°]	**0.006**	0.46	**<0.0001**	−1.06	**<0.0001**	−1.73
Mean elbow angle [°]	**<0.0001**	−0.78	**<0.0001**	1.17	**<0.0001**	2.41
Utilized elbow angle [°]	0.52	−0.07	**<0.0001**	−0.94	**<0.0001**	−0.69
Utilized frontal shoulder angle [°]	0.73	0.02	**<0.0001**	−1.09	**<0.0001**	−1.05
Hand coordination (1.0s)	**<0.0001**	−1.06	**<0.0001**	−1.09	0.75	0.04
Hand coordination (0.2s)	**<0.0001**	−0.93	**<0.0001**	−1.39	0.26	−0.30
Normalized angular momentum X [° s-1]	**0.006**	0.48	**0.0002**	0.59	0.12	0.21
Normalized angular momentum Y [° s-1]	0.12	0.27	0.22	0.34	0.82	0.08
Normalized angular momentum Z [° s-1]	0.37	0.15	**0.01**	0.54	**0.05**	0.43
Stance foot acceleration Y [m s-2]	0.17	0.35	**0.005**	0.58	0.08	0.35
Stance foot acceleration Z [m s-2]	**<0.0001**	0.60	**0.007**	0.53	0.27	−0.06
CoM acceleration X [m s-2]	**<0.0001**	0.76	**0.003**	0.47	0.07	−0.31
CoM acceleration Y [m s-2]	**0.003**	0.53	**0.0003**	0.66	0.30	0.18
CoM acceleration Z [m s-2]	**<0.0001**	1.13	**<0.0001**	1.20	0.68	0.16
Utilized knee angle [°]	0.08	−0.23	**0.02**	−0.56	0.21	-0.34

*P-values are computed using a Wilcoxon Rank-Sum Test. Effect sizes are quantified using Cohen's-d. P-values smaller than 0.05 are highlighted in bold*.

**Table 5:** Group comparison of all performance indicators for walking on a slackline.

	**Beginner/Sportive**		**Beginner/Professional**		**Sportive/Professional**	
**Variable**	***p*-val**	**d**	***p*-val**	**d**	***p*-val**	**d**
Time	**<0.0001**	−1.12	**<0.0001**	−3.18	**<0.0001**	−2.08
Head orientation [°]	**0.04**	−0.57	**<0.0001**	−3.58	**<0.0001**	−1.25
Mean frontal shoulder angle [°]	**0.01**	0.53	**<0.0001**	−1.20	**<0.0001**	−1.71
Mean elbow angle [°]	0.06	−0.46	**<0.0001**	1.69	**<0.0001**	2.32
Utilized elbow angle [°]	0.50	−0.29	**0.002**	−0.84	**0.02**	−0.33
Utilized frontal shoulder angle [°]	0.73	0.00	0.68	0.00	0.51	−0.01
Hand coordination (1.0s)	**<0.0001**	−1.32	**<0.0001**	−1.91	**0.002**	−0.73
Hand coordination (0.2s)	**0.008**	−0.71	**<0.0001**	−1.49	**<0.0001**	−0.87
Normalized kinetic energy [J kg-1]	**<0.0001**	0.96	**<0.0001**	1.84	**0.03**	0.62
Balance energy ratio	**0.003**	−0.74	**0.005**	−0.75	0.37	0.22
Normalized angular momentum X [° s-1]	0.39	0.12	**0.01**	0.71	0.07	0.49
Normalized angular momentum Y [° s-1]	**<0.0001**	1.28	**<0.0001**	2.49	**0.02**	0.61
Normalized angular momentum Z [° s-1]	**0.02**	0.48	**<0.0001**	1.35	**0.005**	0.70
CoM acceleration X [m s-2]	**0.003**	0.53	**0.0002**	1.03	0.83	0.23
CoM acceleration Y [m s-2]	**0.03**	0.49	**<0.0001**	1.53	**<0.0001**	1.02
CoM acceleration Z [m s-2]	**0.0003**	0.89	**<0.0001**	1.49	**0.02**	0.59
Walking speed [m s-1]	**<0.0001**	0.99	**<0.0001**	1.51	0.99	0.10

*P-values are computed using a Wilcoxon Rank-Sum Test. Effect sizes are quantified using Cohen's-d. P-values smaller than 0.05 are highlighted in bold*.

#### 3.2.1. Balance Time on the Slackline

**Figure 7:**
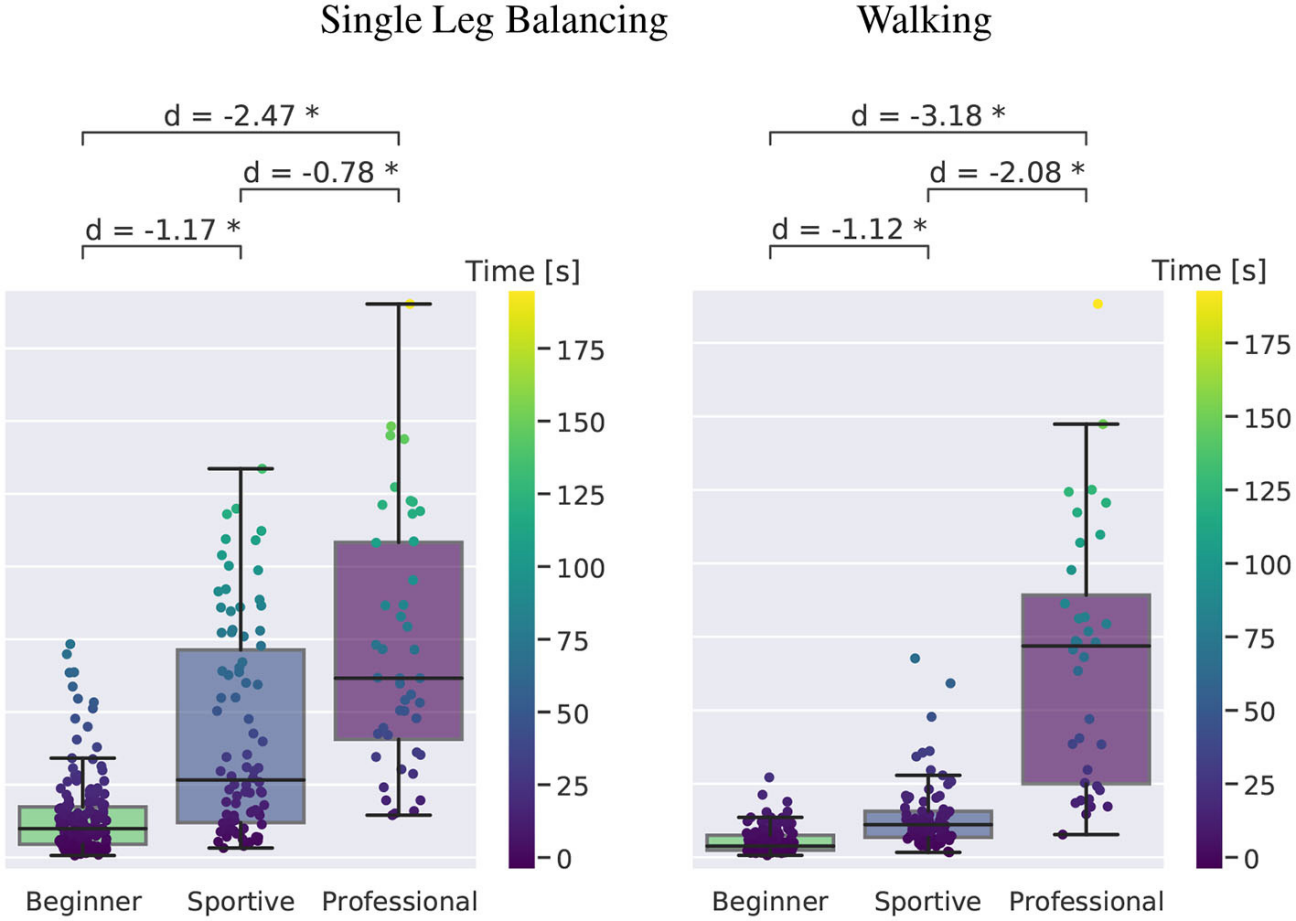
Boxplot of time in balance for single leg slackline balancing for the three subject groups. Boxes contain the inner 50 % of the data and the median is shown. Whiskers are maximum 1.5 times the inner quartile range but end at the last data point which lays inside. Cohen's-d is given for comparison and all values plotted on top with the color following the time, as visualized in the color bar. Significant differences are marked with an asterisk.

Figure 7 shows the grouped time for all trials that were recorded. We found a clear progression in balance time from the beginners to the slackline athletes. Single leg balancing is shown at the left, walking at the right. We quantify effect sizes by means of Cohen's-d (Lakens, 2013). For single leg balancing, beginners manage 14(14) s on average, sportive beginners 42(35) s and professionals 72(42) s. The longest trials are 73, 133, and 190 s, respectively. We see that professional slackliners are able to maintain balance during walking as long as for standing (67(42) s), whereas beginners (5(4) s) and sportive beginners (14(11) s) only manage $\frac{1}{3}$ of the time. Overall, the short slackline setup is beginner friendly and all subjects managed to perform several valid trials. Sportive beginners show significantly longer balance times for both scenarios. They reach similar balance times as experienced slackline athletes for single leg balancing by the end of their first ever slackline session. Walking on a slackline, however is much harder to learn and requires extensive training. Therefore, the gab between the beginner and sportive group to the professional group is much larger.

#### 3.2.2. Quantification of Stability and Recovery Movements

##### 3.2.2.1. Normalized Angular Momentum

**Figure 8:**
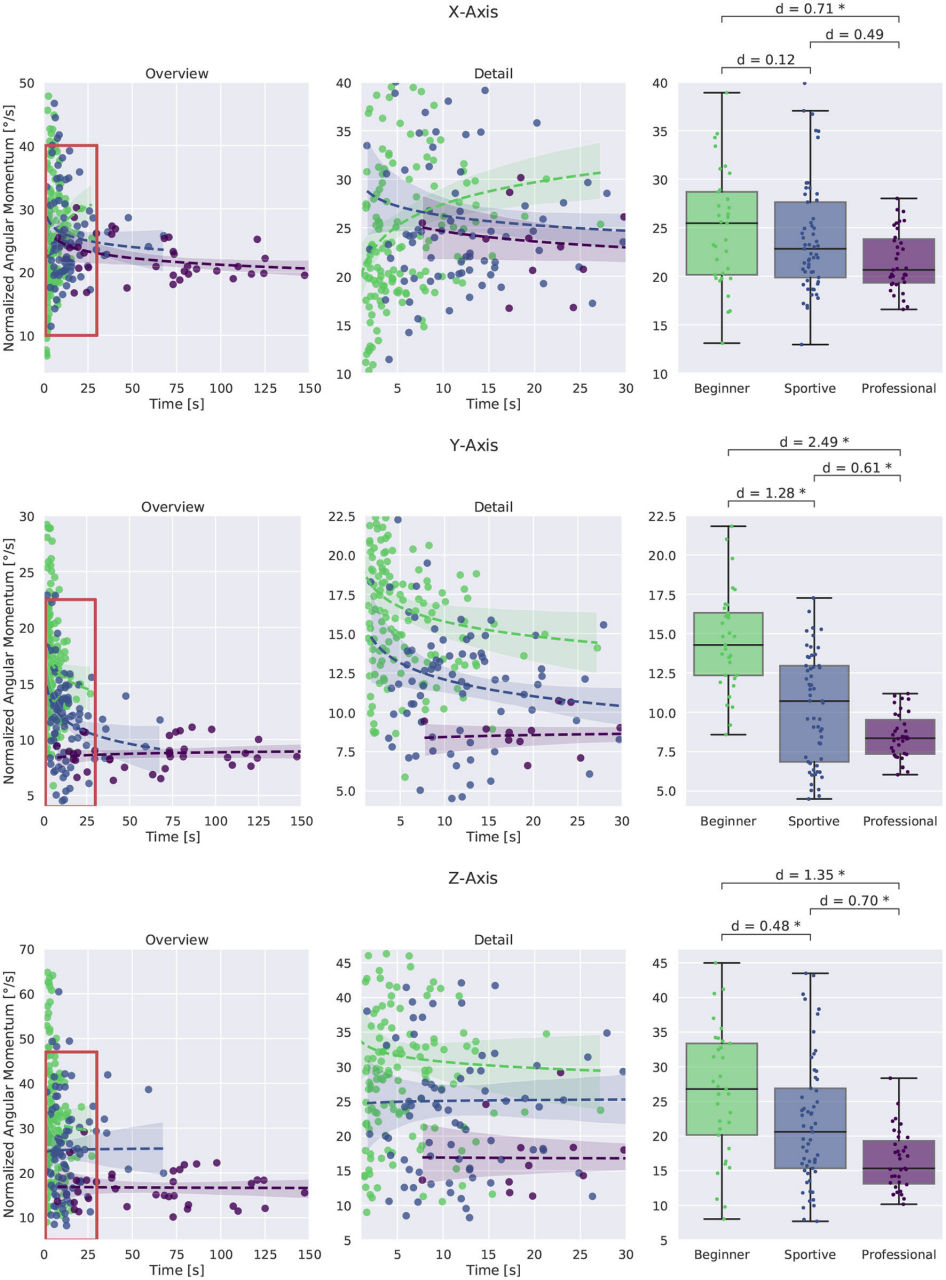
Normalized angular momentum around the three coordinate axes for slackline walking. An overview of all trials is shown at the left, more detail in the middle and box plots for successful trials longer than 8 s at the right. Short trials show larger values, especially in the beginner group. For longer trials minimization of normalized angular momentum around all three coordinate axis becomes important. The professional group shows the smallest values and the least variation between trials. *Corresponds to a significant difference between the groups (*p* < 0.05).

The normalized angular momentum summarizes the active movement and passive rotation of the whole subject. We analyze the three rotation axes separately as shown in Figures 2, 3. Results are similar for the X- and Z-Axis for standing and walking. No group differences were found for the Y-Axis during standing. We therefore only discuss the results for walking in more detail. Figure 8 shows the evaluation for all walking trials. The time in balance is plotted against normalized angular momentum on the left and in more detail in the middle. Box plots for the subject groups are shown on the right. The X-Axis is shown at the top, Y-Axis in the middle row and Z-axis at the bottom. For all three directions we observe a decrease with longer balance trials. We make the following observations:

- The largest normalized angular momentum is observed around the **X-Axis** with about 25 ° s-1. It is the direction that coincides with the instability introduced by the slackline. Beginners show 10 % larger values than sportive beginners and 20 % larger values than the professionals. Values show high variation for shorter and unsuccessful trials justifying their exclusion from the box plot. For successful balancing they converge to 20 ° s-1. This is reasonable since we expect there to be longer stable balancing between recovery movements and smaller recovery movements in general.
- Around the **Y-Axis** beginners tilt forward and backward at a rate of 14 ° s-1. Professionals maintain an upright posture and show about 8 ° s-1 of normalized angular momentum. As mentioned, no differences were found for single leg balancing, suggesting that this direction only becomes relevant during walking.
- Rotation around the **Z-axis** is a key factor in single leg balancing and is equally important for slackline walking. On average, beginners rotate at 26 ° s-1 and sportive beginners at 21 ° s-1, whereas professionals maintain a posture where the arms are perpendicular to the slackline and rotate only at 15 ° s-1.

##### 3.2.2.2. Center of Mass Acceleration

**Figure 9:**
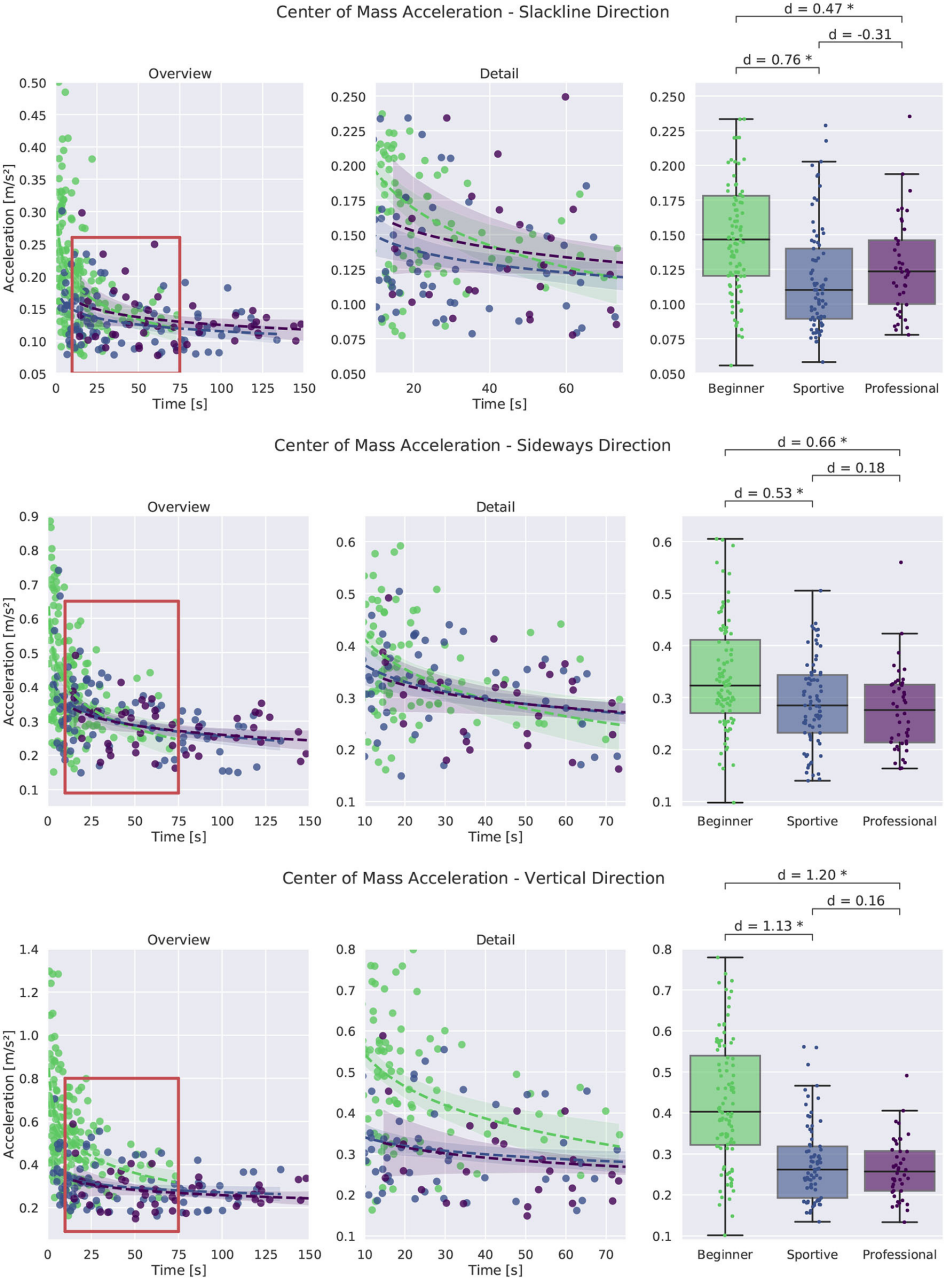
Comparison of Center of Mass acceleration for single leg balancing. An overview of all trials is shown at the left, more detail in the middle and box plots for successful trials longer than 8 s at the right. Significant differences are marked with an asterisk.

Figure 9 shows the CoM acceleration in the three directions for single leg balancing. In all directions we see smaller values with larger times in balance. Both beginner groups show very high accelerations for short and unsuccessful trials (up to 0.8 m s-2). In sideways direction we see the same findings as for the stance foot acceleration. Acceleration values decrease with time and beginners show consistently higher values than professionals. Results are similar for walking. Acceleration in the vertical direction shows the largest difference between the beginner group and the two other groups (*d* = 1.2 and 0.43 m s-1 compared to 0.26 m s-2). For all directions, we see a trend toward reduced CoM acceleration with increasing skill level.

Summarizing we find that normalized angular momentum and CoM acceleration can be used as a metric to measure stability in slackline balancing. Professional slackline athletes are able to maintain an upright posture and their upper body stays perpendicular to the slackline. They do not tilt or move back and forth or up and down. Only balance related arm movement in the sideways direction is performed.

#### 3.2.3. Walking Speed and Energy Ratio

**Figure 10:**
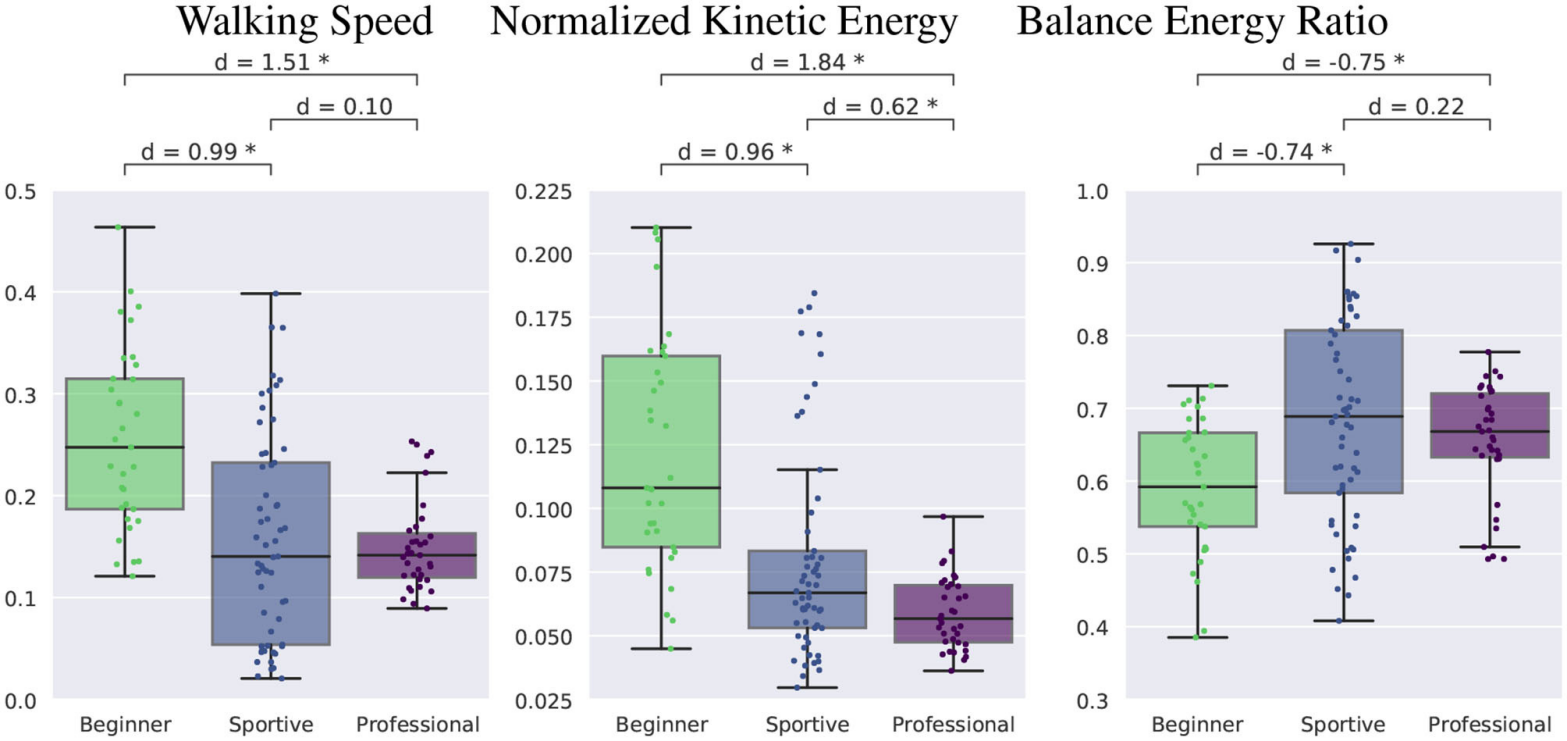
Comparison of walking speed, kinetic energy, and balance energy ratio.

Figure 10 shows the walking speed at the left, the normalized kinetic energy in the middle and the balance energy ratio at the right. We see that beginners attempt to walk faster (0.25 m s-1) and do not focus on balance. Their balance energy ratio is lower than for the sportive beginners and professionals, but their normalized kinetic energy is about twice as large. This result differs from the expectation that beginners would have a higher ratio due to more balance movement. They do not balance enough and try to walk too fast instead. Sportive beginners and professional slackliners walk at similar speeds of 0.15 m s-1 and show a similar balance energy ratio of about 0.7. For flat ground walking this ratio is about 0.4 (Stein, 2021). Overall professionals are more consistent than the two beginner groups.

#### 3.2.4. Balance Specific Skills

##### 3.2.4.1. Movement Coordination

**Figure 11:**
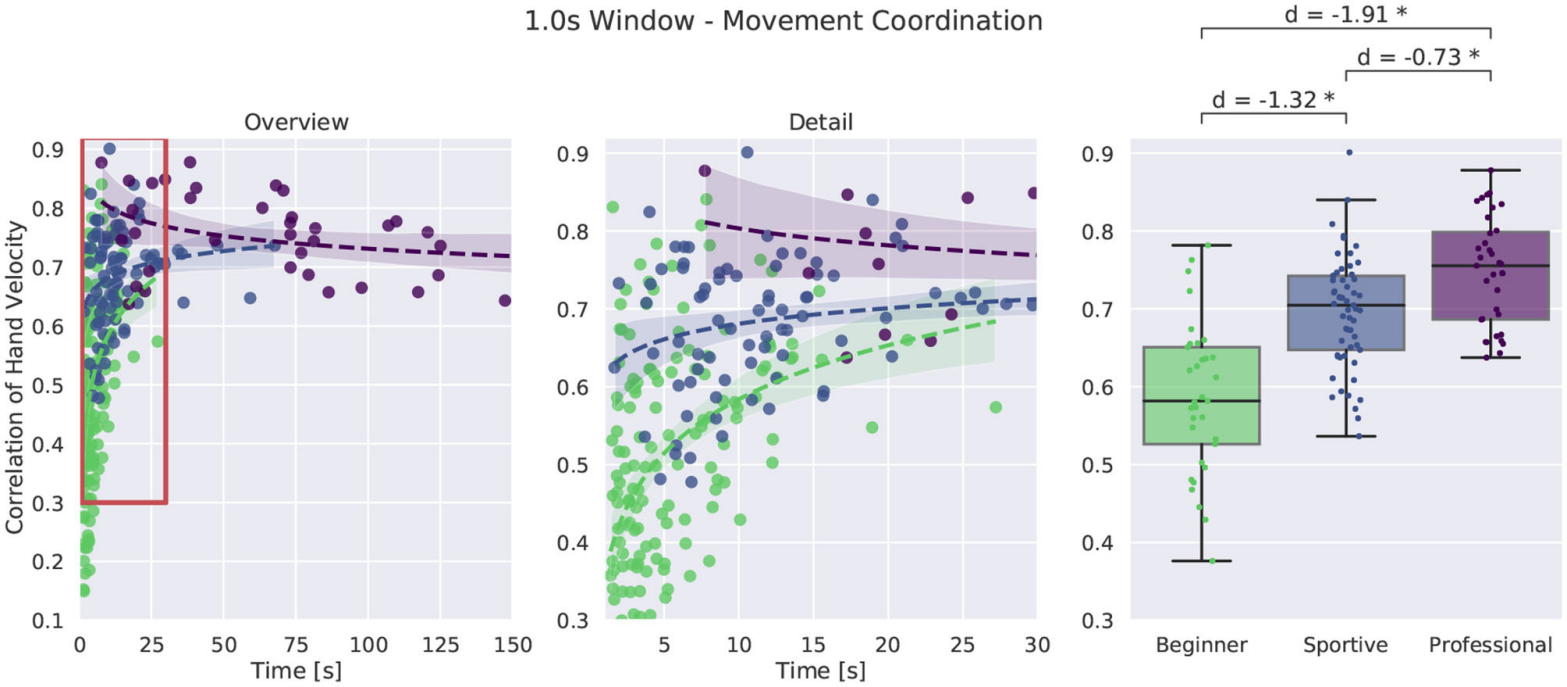
Comparison of movement coordination by means of rolling window correlation during walking. We chose a 1.0 s window. An overview of all trials is shown at the left, more detail in the middle and box plots for successful trials longer than 8 s at the right. *Corresponds to a significant difference between the groups (*p* < 0.05).

For comparison of movement coordination we computed the moving window correlation between the absolute velocity of the hands. We evaluated a time window of 0.2 s and 1.0 s leading to similar results. Figure 11 shows movement coordination values for all walking trials and a 1.0 s window. A larger value represents more coordinated movement. Looking at the box plot at the right, we see that beginner subjects were less coordinated than sportive beginners and professionals outperform the two beginner groups. Especially for short and unsuccessful balance trials, variance is large in the beginner group, whereas the sportive and professional group are much more consistent. For longer trials correlation values converge to 0.7

#### 3.2.5. Slackline Interaction

The interaction force with the slackline is constantly changing and the stance foot is accelerated by the spring-like rubber band. We compare the stance foot acceleration between the subject groups and investigate how the subjects CoM is affected by this. Stance foot acceleration was only evaluated for single leg balancing.

##### 3.2.5.1. Stance Foot Acceleration and Stance Leg Compliance

**Figure 12:**
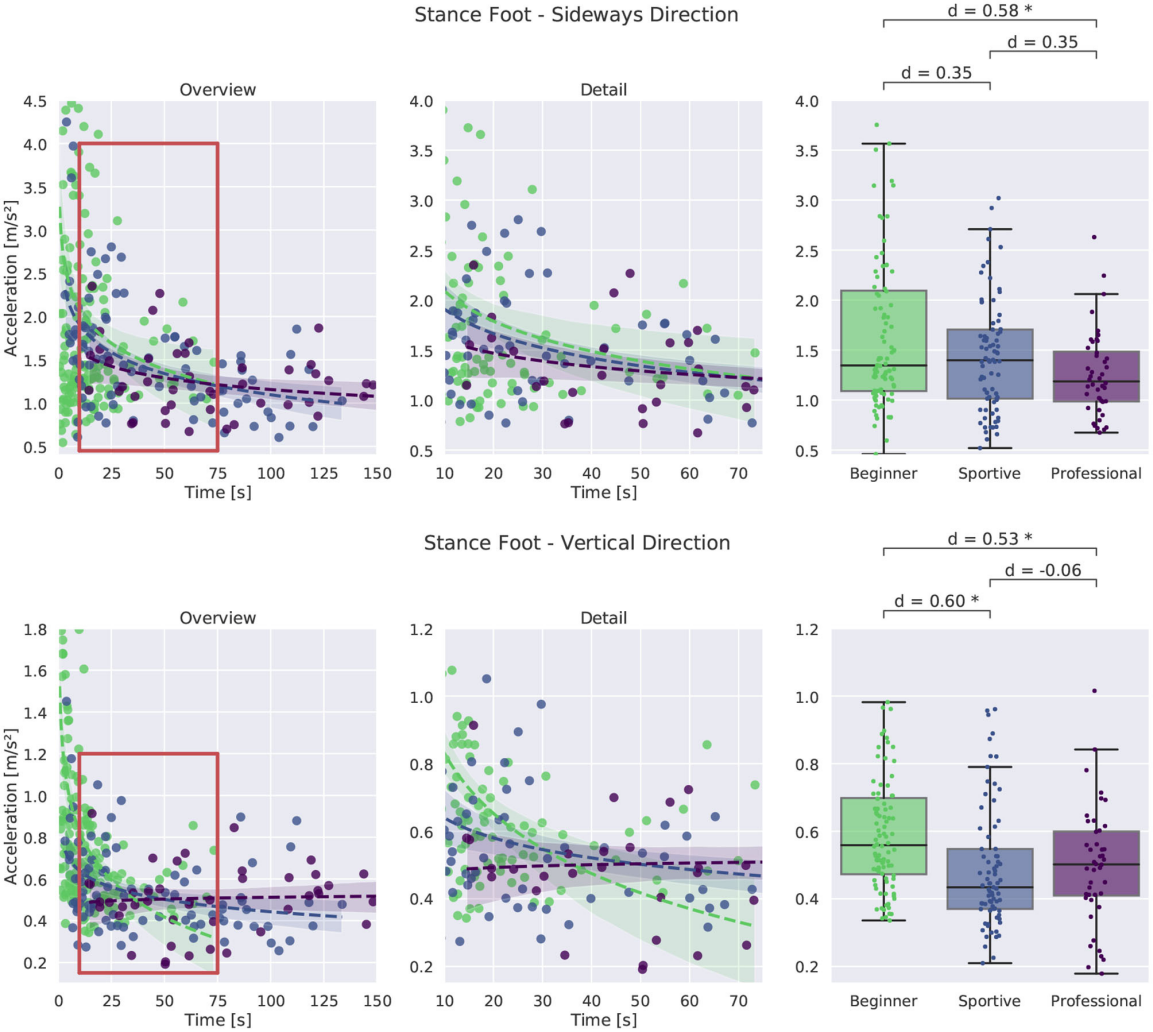
Comparison of stance foot acceleration in vertical and sideways direction for single leg balancing. An overview of all trials is shown at the left, more detail in the middle and box plots for successful trials longer than 8 s at the right. *Corresponds to a significant difference between the groups (*p* < 0.05).

In Figure 12, the foot acceleration in sideways direction is shown at the top and in vertical direction at the bottom. Acceleration in sideways direction is much larger than in vertical direction due to the different masses that are accelerated. In up and down direction, the whole body weight needs to be accelerated by the slackline force, while in sideways direction mainly the leg and feet are accelerated. For the sideways direction we see a clear tendency toward lower values with longer time in balance. Professional slackliners show smaller values when compared to the other groups. The stance foot of beginners is highly accelerated, especially for shorter trials. This is consistent with findings on reduced muscle reflexes and therefore less shaking in the knee (Keller et al., 2011). The shaking sideways leg motion that beginners experience on longer slacklines reduces with training and experience. Correlation between the stance foot and CoM acceleration is high: *r* = 0.83 for both beginner groups and *r* = 0.64 for professional slackliners. We suggest that professional slackliners are able to accelerate their CoM more independently from their stance foot when compared to the two beginner groups.

In vertical direction we find a similar trend with time. Beginners experience high accelerations during short and unsuccessful trials and acceleration is reduced for longer trials. In general, all groups show similarly large variation between trials. The foot of beginners is accelerated between 0.35 and 1.0 m s-2. The sportive group and the professional group show smaller mean values between 0.2 and 0.8 m s-1 suggesting better contact force control.

#### 3.2.6. Balance Strategies

##### 3.2.6.1. Mean Posture

**Figure 13:**
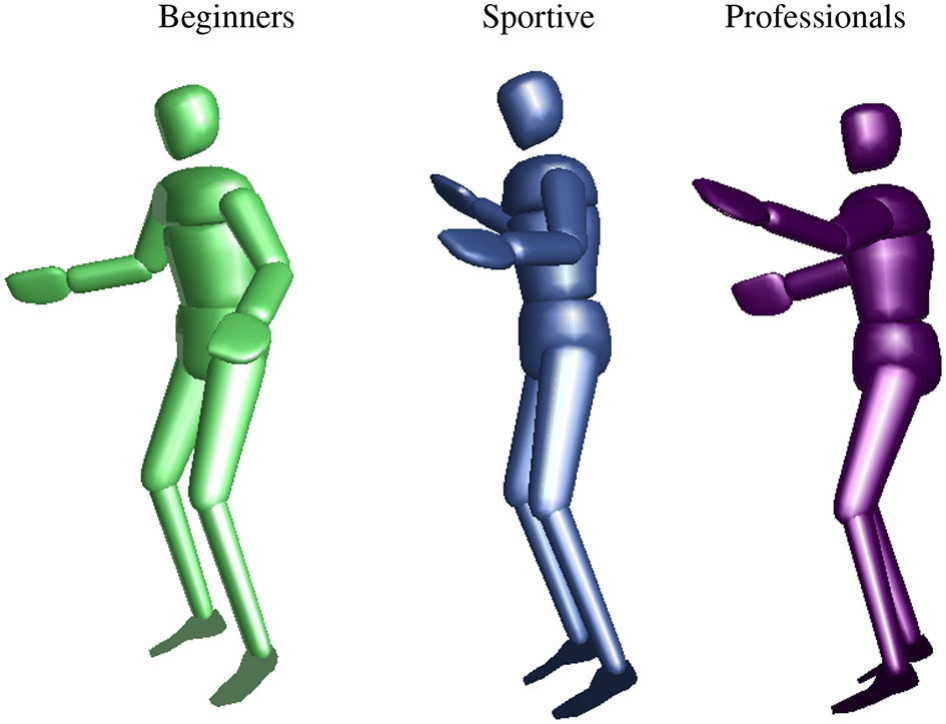
Mean posture during single leg slackline balancing from a side view similar to Figure 3. Beginners are shown at the left, sportive beginners in the middle, and professional slackline athletes on the right. We found differences in head orientation, frontal shoulder angle, and elbow angle.

Figure 13 shows the mean posture for all groups. Leg and trunk configuration are similar, as it is expected, since all trials were cut to stable balancing and the stance leg alternated. Differences are visible for head orientation and arm angles. Professional slackliners have their arms perpendicular to the upper body and elbows extended. They maximize the inertia in the frontal plane. Both beginner groups tend to align their upper arms to the trunk and bend their elbows more. We find 90° compared to 60° for the frontal shoulder joint and 35° compared to 60° for elbow joint.

**Figure 14:**
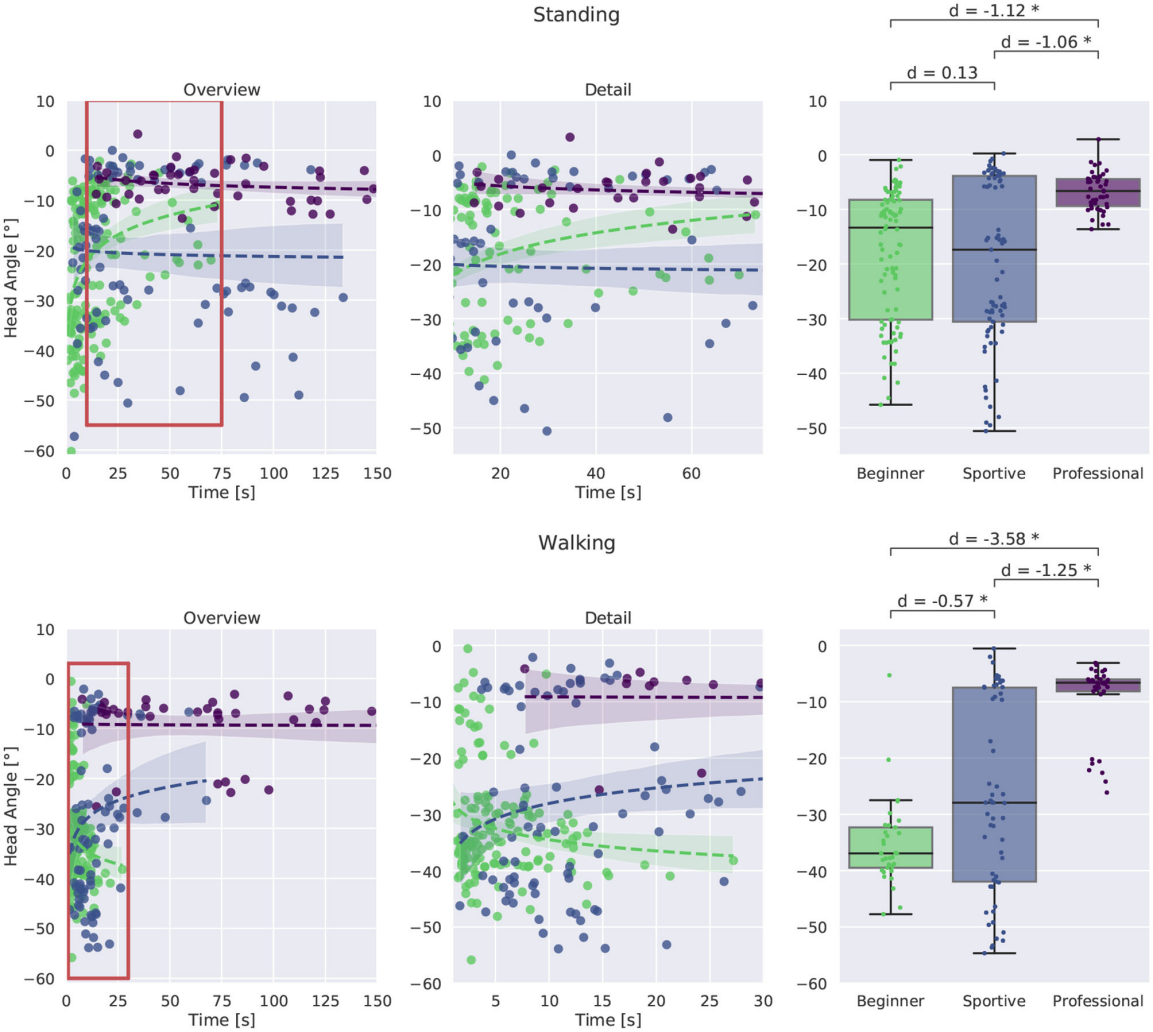
Comparison of head orientation. Standing is plotted at the top, walking at the bottom. Evaluation with respect to time in balance on the slackline is shown in the left and middle column. and between groups. Box plots for successful trials longer than 8 s are plotted at the right. Beginners and sportive beginners show large variation between trials. The professional group is more consistent and manages to maintain a horizontal head orientation. All beginners look down on the slackline during walking. *Corresponds to a significant difference between the groups (*p* < 0.05).

Details on the head orientation are shown in Figure 14. In the left column we plotted the time in balance on the slackline against the head angle for standing and walking. More detail is shown in the middle and box plots comparing the groups at the right. All professional slackline athletes maintain a similar head orientation of about -5° with respect to the horizontal plane for standing and for walking. They are consistent throughout trials, whereas the two beginner groups are varying from trial to trial, especially for unsuccessful and short trials. All beginners look down at the slackline during the walking task, suggesting that they are insecure about the slackline position and ensure correct foot placement. The sportive group varies between looking at the feet (-45°), at the end of the slackline (-25°) and horizontally (-5°).

##### 3.2.6.2. Arm Movement

We compare the amount of movement by means of variation around the mean joint angle. The professional group uses more of the possible shoulder range of motion, suggesting that they are stable in a greater variety of poses. We found a variation of 29° compared to 19° in the two beginner groups for the frontal shoulder. They are, for example, able to point both arms in the same direction during a recovery movement and come back to the regular t-pose balancing, while beginners and sportive beginners are not able to perform this recovery movement and fall. Furthermore we found that beginners and sportive beginners used less of their range of motion in the elbow joint 12° compared to 16°. Again, this suggests a greater variety of poses in which the professional group is able to maintain balance.

## 4. Summary and Conclusion

We analyzed slackline balancing for beginners that had never balanced on a slackline before and compared them to professional slackline athletes. Furthermore, all study participants performed a static balance test. As a first result we found that trained slackliners performed very well in the static balance test, whereas the beginner group showed a larger variance in the time they managed to balance. We therefore decided to divide the beginner group into a balance-experienced, sportive group that performed similarly to the professional group and a balance-inexperienced, beginner group. Especially the single leg scenario with eyes closed allowed us to distinguish two groups based on whether the subject managed to maintain balance for 30 s. We found strong correlation between the time in balance with eyes closed and the time that a subject was able to balance on the slackline. The balance-experienced beginners group managed to maintain balance on the slackline about three times longer than the balance-inexperienced group. We conclude that static balance can be predictor for slackline balance performance, especially single leg balancing with eyes closed.

We then defined performance indicators for slackline balancing and analyzed over 300 balancing trials of 20 participants. Normalized angular momentum and CoM acceleration allow us to quantify how stable and controlled a subject is while balancing. They summarize the amount and intensity of recovery (arm) movements. Reduced rotation around the vertical axis allows professional slackliners to maintain an upright posture and focus their arm movement in the frontal plane, whereas beginners and sportive beginners show larger movement in all three directions. Differences in pose and strategy were found between the professional group and the beginner and sportive group. Posture and movement were similar for the beginner and sportive beginner group. Professional slackline athletes perform most of the balance related movement in the elbow joint and maintain a perpendicular arm configuration. They show a 50 % larger utilized range of motion in the shoulder and elbow joint. We conclude that they are more versatile in performing balance movements and can maintain balance in a larger set of postures. Beginners are stable only in some postures, but fall in others, where professionals are still able to balance.

We analyzed the interaction with the slackline by means of stance foot acceleration in relation to CoM acceleration. In horizontal direction we found a large correlation between the values for all participants. Professional slackliners show reduced values for both performance indicators. This confirms findings in the literature of adjusted muscle reflexes from slackline training. In vertical direction the sportive group and the professional group show reduced values for CoM acceleration when compared to beginners. Both findings suggest, that control of the stance foot acceleration is a key factor to successful slackline balancing.

We suggest, that the sportive beginners group does have better proprioception than the balance-inexperienced beginners. Findings for hand coordination support this claim. Maintaining an upright head position with respect to gravity is crucial for the vestibular system to function properly (Goldberg and Fernandez, 1984). Indeed, the professional group consistently maintains a horizontal head orientation. This can also be linked to visual feedback. We assume that their gaze is fixed to a point, as it was shown by Schaerli et al. (2013), whereas, especially during walking, beginners need to confirm their foot placement.

Based on the slackline balance research presented in this work there are many possible future studies. The balance indicators for slackline balancing are by no means complete and different metrics can reveal additional skills that allow professional athletes to balance. For balance training, it is interesting to understand how the performance indicators change over the course of a longer balance training program.

The study is limited in two regards: It has yet to be shown that the performance indicators found for slackline balancing are also valid during other balance tasks. This can be a very similar task such as tightrope walking or the tandem walk, but also a more general balance related sport like snowboarding or surfing. Further, an analysis of the tandem stance could not be performed using the methodology of the study. The task was to hard to achieve for beginners and the ratio of valid trials longer than 8 s was too low for an unbiased analysis.

## Data Availability Statement

The raw data supporting the conclusions of this article will be made available by the authors, without undue reservation.

## Ethics Statement

The studies involving human participants were reviewed and approved by the Ethics Committee of the Faculty of Behavioral and Cultural Studies of Heidelberg University according to the Helsinki Declaration (AZ Mom 2016 1/2-A1, 2016 with amendment 2019). The patients/participants provided their written informed consent to participate in this study.

## Author Contributions

KS and KM: conceptualization. KS: formal analysis, investigation, and writing—original draft. KM: funding acquisition, project administration, supervision, and writing—review and editing. Both authors have read and agreed to the published version of the manuscript.

## Funding

Funding by the Carl Zeiss Foundation within the Heidelberg Center for Motion Research is gratefully acknowledged.

## Conflict of Interest

The authors declare that the research was conducted in the absence of any commercial or financial relationships that could be construed as a potential conflict of interest.

## Publisher's Note

All claims expressed in this article are solely those of the authors and do not necessarily represent those of their affiliated organizations, or those of the publisher, the editors and the reviewers. Any product that may be evaluated in this article, or claim that may be made by its manufacturer, is not guaranteed or endorsed by the publisher.
